# Highly Entangled Hydrogels by Photoiniferter‐Mediated Polymerization

**DOI:** 10.1002/anie.202421970

**Published:** 2025-02-21

**Authors:** Gavin Irvine, Konstantinos Myronidis, Fulvio Pinto, Maciej Kopeć

**Affiliations:** ^1^ Department of Chemistry University of Bath, Claverton Down Bath BA2 7AY UK; ^2^ Department of Mechanical Engineering University of Bath, Claverton Down Bath BA2 7AY UK

**Keywords:** polymer networks, entanglements, hydrogels, rheology, RAFT

## Abstract

We report the synthesis of ultra‐high molecular weight (UHMW) poly(*N,N*‐dimethylacrylamide) (PDMAm) hydrogels with extremely low crosslinking densities by trithiocarbonate photoiniferter‐mediated reversible deactivation radical polymerization (RDRP). Fixing the photoiniferter to crosslinker ratio and gradually increasing the targeted degree of polymerization (*DP*_target_) allowed for simultaneous control over the crosslinking density and the average molecular weight (*M*_n_) of the primary chains, both below and above the critical molecular weight of entanglement (*M*_c_). Interestingly, a plateau in storage moduli (*G’*) was observed for UHMW PDMAm hydrogels with a sufficiently high *DP*_target_ (>5,000), indicating a transition to the entanglement‐dominated regime, with no contribution from crosslinks to the overall modulus, thus indicating the formation of highly entangled hydrogels. These hydrogels exhibit enhanced properties such as high toughness and resistance to swelling despite their vanishingly small crosslinking densities. Furthermore, even when equipped with cleavable crosslinkers, the UHMW PDMAm hydrogels resist degradation due to dense entanglements which act as transient crosslinks preventing the gels from swelling, while sparse covalent crosslinks help to maintain their structural integrity and avoid chain disentanglement. This approach allows simple synthesis of elastic and tough hydrogels with a well‐defined structure and tuneable contributions from both crosslinks and entanglements.

## Graphical abstract

Ultra‐high molecular weight (UHMW) poly(N,N‐dimethylacrylamide) hydrogels with extremely low crosslinking densities were synthesized by photoiniferter‐mediated reversible deactivation radical polymerization (RDRP). The resulting hydrogels contain high density of trapped entanglements which impart enhanced properties such as high toughness and resistance to swelling despite the vanishingly small crosslinking densities.

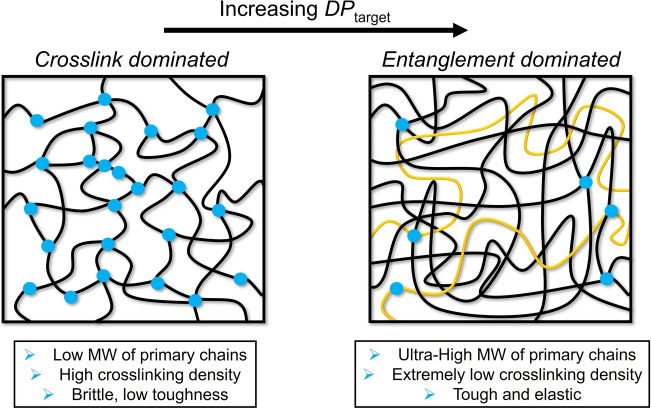

## Introduction

The unique physical properties of polymer networks, such as rubberlike elasticity or ability to swell in solvents, are a direct result of their crosslinked structure. Controlling the crosslinking density is therefore the primary way to tune virtually any property of thermosets, elastomers and (hydro)gels.[^1^, ^2^, ^3^, ^4^, ^5^, ^6^] Indeed, high crosslinking density increases the elastic modulus of a network and decreases its swelling capacity. However, it will also reduce toughness of the material, making it more brittle. Furthermore, real polymer networks contain various defects such as spatial inhomogeneities, topological loops, dangling ends and entanglements which also affect the mechanical properties but are much more difficult to control and quantify than crosslinking density.[^3^, ^7^, ^8^, ^9^, ^10^, ^11^, ^12^, ^13^, ^14^, ^15^, ^16^]

Strategies adopted to resolve the conundrum between the strength and toughness in polymer networks include preparation of double network hydrogels, slip‐ring or mechanopore‐containing crosslinks, ‘ideal’ (e.g. tetra‐PEG) networks, or highly entangled hydrogels, each employing a different stress relaxation mechanism to allow energy to dissipate during deformation.[^17^, ^18^, ^19^, ^20^, ^21^] Highly entangled hydrogels (also called tanglemers) have been recently proposed by Suo^[22]^ and Miyata^[23]^ and realised by synthesis of polyacrylamide (PAAm) hydrogels where physical entanglements significantly outnumbered chemical crosslinks. This was ensured by performing free radical polymerization (FRP) at extremely low loadings of both the initiator (to maximize the molecular weight) and crosslinker (to minimize the crosslinking density). The resulting hydrogels exhibited resistance to fatigue and were highly tough due to the entanglements acting as effective physical crosslinks, while the extremely low crosslinking density ensured the structural integrity of the hydrogels but did not negatively influence their toughness.[^22^, ^23^, ^24^, ^25^, ^26^, ^27^]

Other recent examples of highly entangled hydrogels involved crosslinking of pre‐synthesized, ultra‐high molecular weight (UHMW) polymers^[28]^ or proteins,[^29^, ^30^] well above the critical molecular weight of entanglement (*M*_c_).[^31^, ^32^] However, crosslinking of pre‐synthesized UHMW polymers may result in lower‐than‐expected crosslinking density due to their high viscosity and steric hindrance, while crosslinking under FRP conditions inevitably leads to formation of spatial heterogeneities whose contribution, although likely diminished at very low crosslinker loadings, cannot be ruled out.[^7^, ^8^, ^33^]

On the other hand, reversible deactivation radical polymerization (RDRP) techniques such as atom transfer radical polymerization (ATRP), or reversible addition‐fragmentation chain transfer (RAFT) polymerization have been routinely used to synthesize polymer gels/networks. It is well established that due to the controlled growth of uniform chains, RDRP techniques produce more homogeneous networks, devoid of spatial inhomogeneities.[^34^, ^35^] However, contrary to FRP‐made gels, the typical molecular weights of the primary chains in networks made by RDRP are rather low, with targeted degrees of polymerization (*DP*_target_) in the range of 50–500.[^34^, ^35^, ^36^, ^37^, ^38^, ^39^, ^40^, ^41^, ^42^, ^43^] This is below the *M*_c_ of commonly used poly(meth)acrylates or polyacrylamides,[^31^, ^32^, ^44^, ^45^] making entangled RDRP gels/networks an underexplored class of polymer materials. Very recently, strategies to introduce entanglements post‐gelation into RAFT networks by supramolecular templating^[14]^ or *in situ* formation of an interpenetrated polymer network (IPN) structure^[46]^ have been reported by the Zhukhovitskiy and Konkolewicz groups, respectively.

This scarcity of examples of RDRP networks in the entangled regime is partially due to the traditional difficulties in synthesizing UHMW polymers by RDRP, caused by an increased rate of termination at high conversions.[^47^, ^48^, ^49^, ^50^, ^51^] Overcoming this limitation, Sumerlin *et al*. reported the facile synthesis of UHMW poly(*N,N*‐dimethylacrylamide) (PDMAm) (*M*_n_>1×10^6^ g mol^−1^) by photoiniferter‐mediated RDRP^[52]^ in aqueous media using a trithiocarbonate‐based photoiniferter and no external radical source.[^53^, ^54^, ^55^] This allowed preparation of well‐defined polymers with *DP*_target_ over 85,000, reaching high monomer conversions within minutes, and was later applied to the synthesis of UHMW polyacrylates[^56^, ^57^] and polystyrene.^[56]^ RDRP‐synthesized UHMW polymers were also explored to study relaxation dynamics in vitrimers^[58]^ and to prepare self‐assembled microparticles.^[59]^

We sought to utilize this synthetic approach in the presence of a crosslinker to enable rational design of highly entangled hydrogels by systematically decreasing the crosslinking density and increasing *M*_n_ of the primary chains. By leveraging the uniform, ‘living’ chain growth in RDRP methods, both *M*_n_ and crosslinking density could be simultaneously controlled by fixing the photoiniferter to crosslinker molar ratio while changing *DP*_target_ (i.e., the monomer to photoiniferter ratio). This would allow the transition from unentangled to entangled network when *M*_n_>*M*_c_, and gradually decrease the crosslinking density by simply adjusting the *DP*_target_.

Indeed, according to the Edwards tube model, the modulus of the entangled polymer network can be approximated as shown in equation 1:

(1) $$G=G_{x}+G_{e}\approx \rho RT\left(\frac{1}{M_{x}}+\frac{1}{M_{e}}\right)$$

Where *G*_x_ and *G*_e_ are moduli contribution from crosslinks and entanglements, *M*_x_ is the average molecular weight between crosslinks, *M*_e_ is the average molecular weight between entanglements, and ρ is polymer density.^[60]^ It follows that at sufficiently high *M*_x_ (and hence, *DP*_target_), the contribution of the crosslinks should become negligible, and modulus should only depend on *M*_e_, indicating the formation of highly entangled hydrogels.

## Results and Discussion

**Scheme 1:**
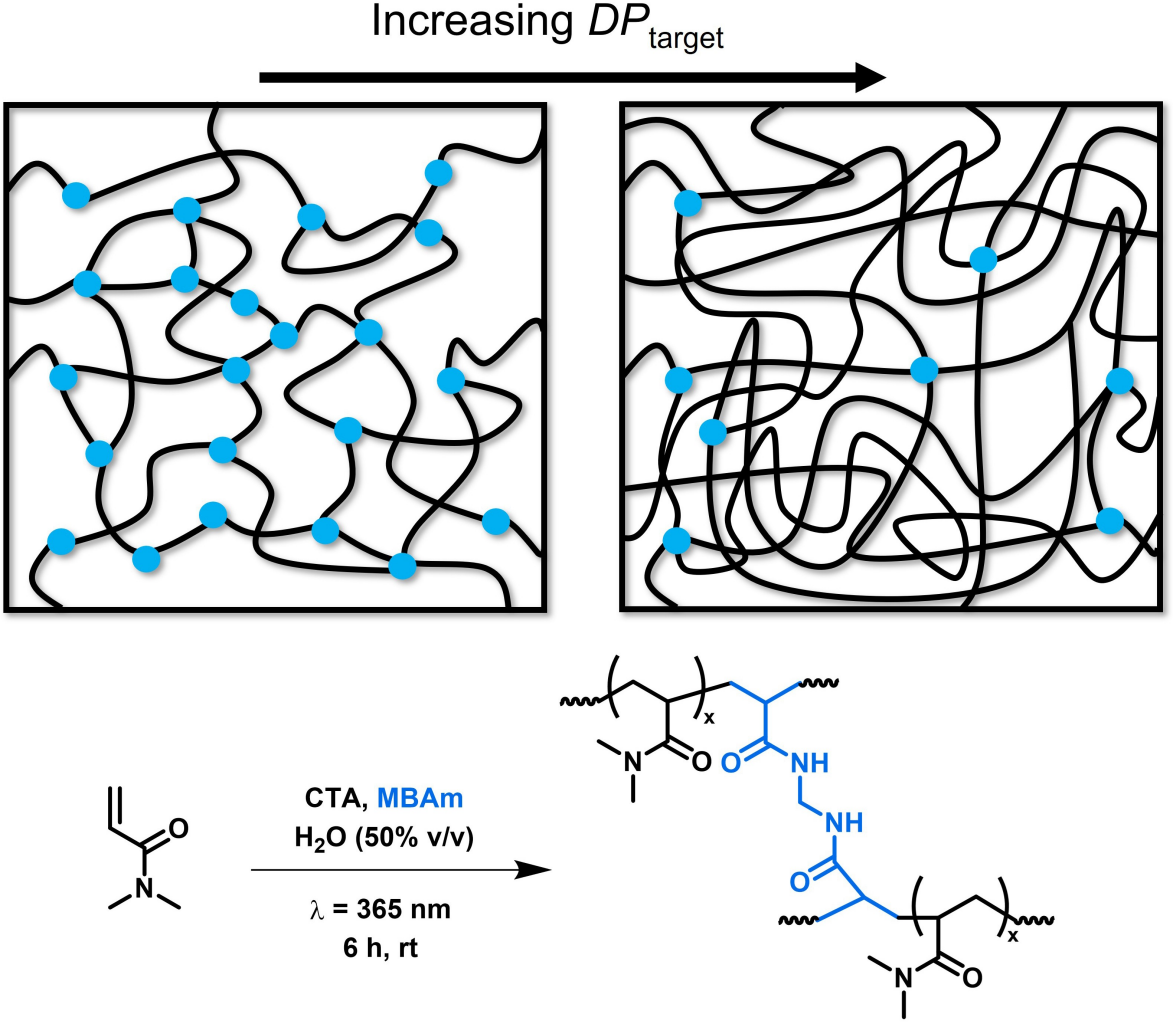
Representation of the synthetic procedure used for the preparation of UHMW PDMAm‐MBAm hydrogels in this work alongside a schematic visualisation of how entanglement density increases, and crosslinking density decreases as *DP*_target_ is increased at a fixed MBAm/CTA ratio.

**Synthesis and rheology of UHMW hydrogels**. The UHMW PDMAm hydrogels were prepared by photoiniferter‐mediated RDRP (Scheme 1). The polymerization was initiated by direct photocleavage of the photoiniferter (or chain transfer agent, CTA), 2‐cyano‐2‐propyl dodecyl trithiocarbonate at λ_max_=365 nm. A [DMAm] : [CTA] ratio (i.e., *DP*_target_) ranging from 500 to 100,000 was used with a fixed ratio of *N,N’*‐methylenebisacrylamide (MBAm) crosslinker relative to the CTA ([MBAm] : [CTA]=2–10) at a DMAm/water ratio of 1 : 1 (v/v, i.e. [DMAm]_0_=4.73 M). At a fixed [MBAm] : [CTA] ratio, increasing *DP*_target_ will increase both the *M*_n_ of the primary chains and *M*_x_.

**Figure 1:**
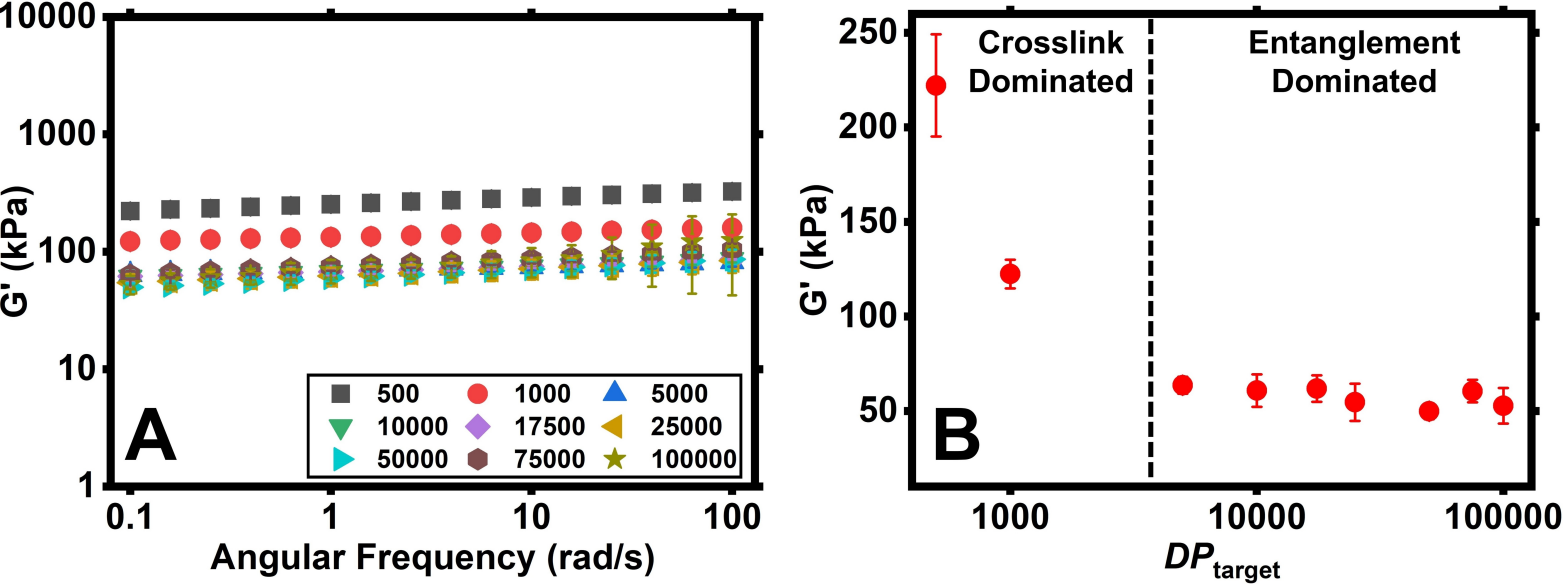
Oscillatory rheology frequency sweeps of UHMW PDMAm hydrogels with [MBAm] : [CTA]=10 and varying *DP*_target_ (**A**) and the storage modulus (*G’*) data at 0.1 rad/s for each *DP*_target_ (**B**). All measurements were performed in triplicate.

Figure 1A shows the shear storage moduli (*G’*, kPa) measured by oscillatory rheology for the as prepared PDMAm hydrogel discs with [MBAm] : [CTA]=10. Each hydrogel had a thickness of c.a. 7 mm and a gel fraction >95 % (Table S1). All hydrogels showed constant storage moduli values within the frequency range of 0.1–100 rad/s, indicating no significant frequency dependence, whereas low values of loss moduli (*G”*) and phase angles well below 45° confirmed that the hydrogels behaved more elastically than viscously (Figure S1).

Interestingly, for the PDMAm hydrogels with [MBAm] : [CTA]=10, a plateau in moduli values at 0.1 rad/s (corresponding to the rest state at long timescales) was observed for the range of *DP*_target_ between 5000–100,000 (Figure 1B). For example, the hydrogel with a *DP*_target_ of 5000 has a modulus value of 59.20±4.92 kPa while the sample with a *DP*_target_ of 100,000 (a 20‐fold decrease in crosslinking density) has a modulus value of 62.01±8.09 kPa. Hydrogels with lower crosslinker content ([MBAm] : [CTA]=2–8) showed analogous behaviour, with similar moduli values recorded above 5000 *DP*_target_ (Figure S2). However, for the [MBAm] : [CTA]=2 series, the reduction in *G’* in the crosslink‐dominated region is not observed due to the low initial crosslinking density.

This observable plateau is clearly in agreement with equation 1, as *M*_x_ in a PDMAm hydrogel with a *DP*_target_ of 10,000 would be 10 times lower than one with a *DP*_target_ of 100,000, yet they have nearly identical *G’* values. This suggests that only *M*_e_ contributes to the overall modulus for higher *DP*_target_ hydrogels, with no measurable contribution from the permanent chemical crosslinks. Therefore, by using RDRP to gradually increase the *DP*_target_ of the primary chains, the transition between the crosslink‐dominated and entanglement‐dominated modulus regimes can be observed and used to assess the formation of highly entangled hydrogels. A similar relationship between the modulus and crosslinking density was observed by Miyata *et al*. for PAAm hydrogels synthesized by FRP.^[23]^ However, in FRP the presence of entanglements cannot be easily controlled, whereas in RDRP increasing the *DP*_target_ will both lower the crosslinking density and gradually introduce entanglements, affording the observation of the transition from unentangled to entangled network.

In order to quantify this transition, *M*_e_ was determined for uncrosslinked PDMAm in melt to be 15,400 g mol^−1^ (see Figure S3).^[61]^ The *M*_c_/*M*_e_ ratio is typically in the range 1‐3.5, and often assumed as *M*_c_=2*M*_e._[^31^, ^32^] However, *M*_c_ is only characteristic for a melt, and will increase in solution according to eq. 2:

(2) $$M_{c,\;sol}=\;\frac{M_{c}}{\varphi^{\alpha }}$$

where *M*_c,sol_ is the critical molecular weight in solution, *φ* is the polymer volume fraction and α is the dilution coefficient equal to 1 or 4/3.^[32]^ Therefore, the entanglements in solution start to form at a higher *M*_c_. For our preparation conditions, *φ*=0.47, and assuming α=1 and *M*_c_,=2*M*_e_, *M*_c,sol_ can be calculated to 65,500 g mol^−1^ and rising to 147,400 g mol^−1^ if α=4/3 and *M*_c_,=3.5*M*_e_ are assumed. Thus, the hydrogels with *DP*_target_=500 are below the entanglement limit (i.e., *M*_n_<*M*_c,sol_) which is reached only at *DP*_target_≥1000. However, *DP*_target_=1000 is still well within the crosslink‐dominated regime in Figure 1B as crosslinking density has not been sufficiently decreased to reach the modulus plateau.

**Figure 2:**
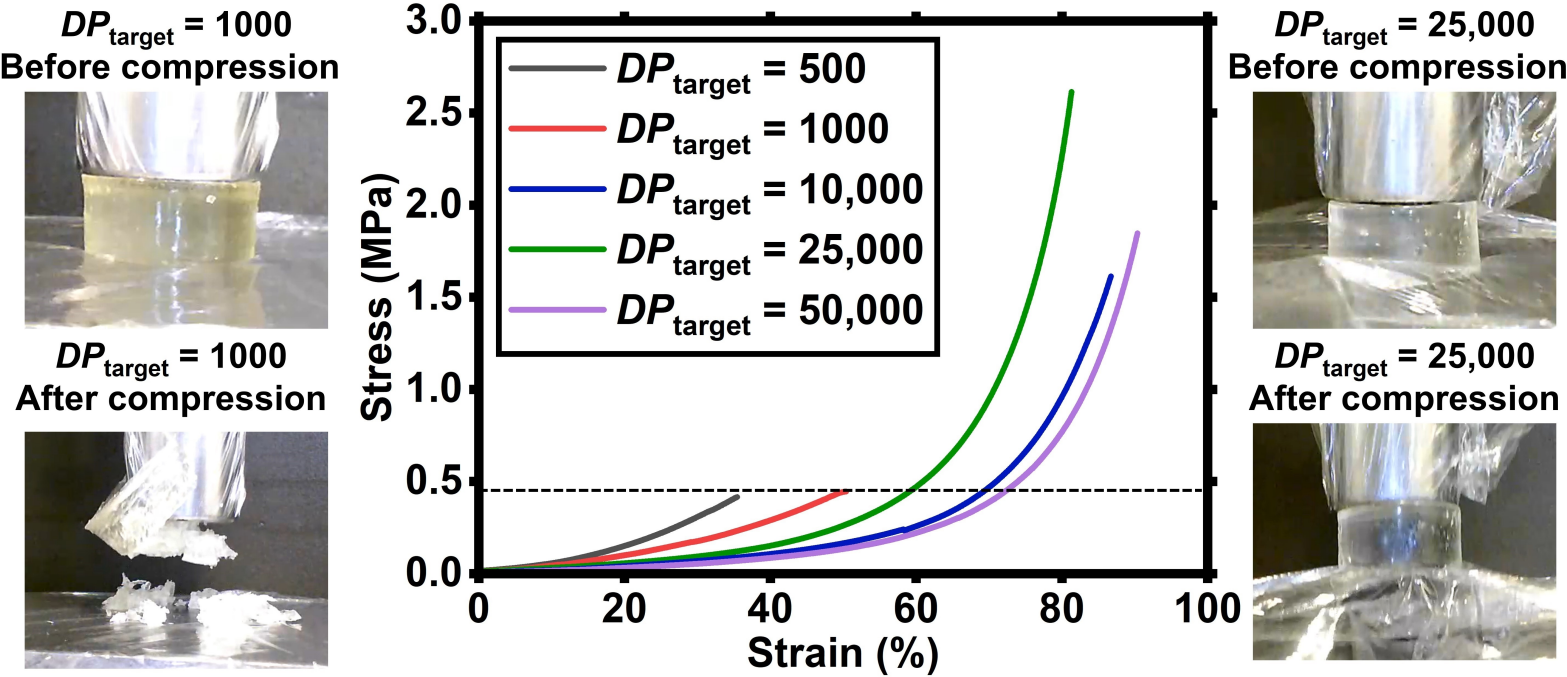
Compressive stress (MPa) vs strain (%) for PDMAm‐MBAm hydrogels with *DP*_target_=500–50,000 hydrogels at 1 kN force applied with a displacement of 7–8 mm. Images of the two hydrogels with *DP*_target_=1000 and 25,000 both before (top) and after (bottom) compression are shown.

**Compression tests**. To assess the influence of entanglements on the toughness of the PDMAm hydrogels, compression tests of as‐synthesized samples were carried out using a mechanical testing instrument (Instron Universal Test Frame 3369) equipped with a 1 kN load cell and tested with a compression velocity of 1 mm min^−1^. Five PDMAm hydrogels were tested, in the *DP*_target_ range between 500 and 50,000. The stress‐strain curves from this analysis are shown in Figure 2 along with the images of two samples, one from the crosslink‐dominated region (*DP*_target_=1000) and one from the entanglement‐dominated region (*DP*_target_=25,000) before and after compression (see also Figures S4 and S5 as well as Movies S1–S5).

The compressive test results from Figure 2 show a clear difference between the crosslink‐dominated and entanglement‐dominated hydrogels. The hydrogels with lower *DP*_target_ (i.e. 500 and 1000) behave like brittle solids, showing a catastrophic failure at around 50 % of the compressive strain (indicated with a dotted black line in the Figure), while the *DP*_target_=10,000 to 50,000 hydrogels present a more viscoelastic behaviour, being resistant to compression and recovering their shape even when more than 80 % of compressive strain is applied. However, the *DP*_target_=10,000 still undergoes partial failure and cracks (as seen in Figure 2 with the small drop at around 58 % strain and in Movie S3) while the *DP*_target_=25,000 and 50,000 samples stay intact throughout the test. This change in the compressive behaviour also affects the compressive strength of the hydrogels, with the *DP*_target_=25,000 sample reaching a value of maximum stress five times higher than the *DP*_target_=1000 sample (2.615 MPa and 0.446 MPa, respectively).

Notably, the hydrogel with *DP*_target_=1000 has *M*_n_>*M*_c, sol_ as well as a crosslinking density of 10^−2^ (vs monomer), as indicated by its relatively low *G’* (114.54±4.67 kPa, Figure 1). However, as it does not reach the modulus plateau (43.87±3.89 – 62.01±8.09 kPa), the contribution from the crosslinks is still too pronounced and the hydrogel is brittle. Only when the crosslinking density becomes vanishingly small (i.e., 4×10^−4^ vs monomer for the 25,000 *DP*_target_ hydrogel) with no detectable contribution to modulus, does the toughness of the hydrogel significantly increase. Recently, Konkolewicz *et al*. demonstrated an increased resistance to compressive stress of otherwise unentangled networks made by RAFT, after introducing entanglements by in situ formation of an IPN structure.^[46]^ Here, entanglements are incorporated into a single network hydrogel by increasing the *M*_n_ of the primary chains and, critically, the *M*_x_ to reach the plateau modulus.

**Figure 3:**
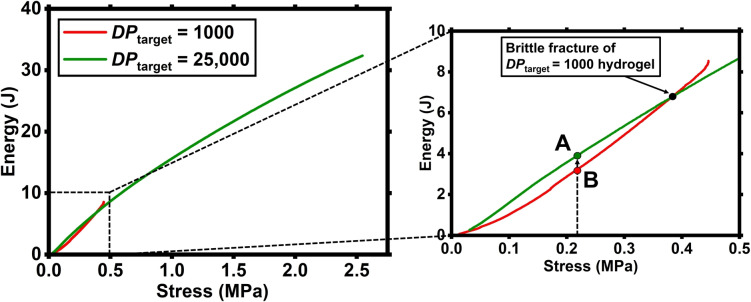
(Left) energy absorption diagrams for the 1000 and 25,000 *DP*_target_ hydrogels. (Right) zoomed in region between 0 and 0.5 MPa showing the difference in trend between the two hydrogels up to the point of fracture of the *DP*_target_=1000 sample.

The effect of the reduction in the crosslink density and the consequent increase of toughness was further examined by comparing the energy absorption diagrams for the 1000 and 25,000 *DP*_target_ hydrogels (Figure 3). Indeed, by calculating the energy absorbed during the compressive tests (i.e., the area under the compressive stress‐strain curve) and plotting it as a function of the applied stress, it is possible to assess how the change in the internal structure of the polymer affects the stress redistribution mechanism for the two hydrogels.^[62]^

As can be seen from the two curves, by increasing the *M*_n_ of the primary chains and decreasing crosslinking density, the behaviour of the hydrogel becomes dominated by the entanglements, leading to an increase in the total absorbed energy by more than 250 % (from 8.52 J to 32.36 J). This change in behaviour is also clear when we compare the different trends of the two hydrogels up to the brittle fracture of the *DP*_target_=1000 sample. Indeed, for a given value of applied stress, the energy absorbed by the *DP*_target_=25,000 sample (point A) is 25 % higher than the energy absorbed by the *DP*_target_=1000 sample (point B). This behaviour is consistent throughout the entire curve until the brittle failure of the *DP*_target_=1000 sample, indicating that the *DP*_target_=25,000 sample is characterized by more optimized stress redistribution properties under the same loading conditions. This reflects its higher energy absorption and a greater capacity in terms of toughness and impact resistance.

**Figure 4:**
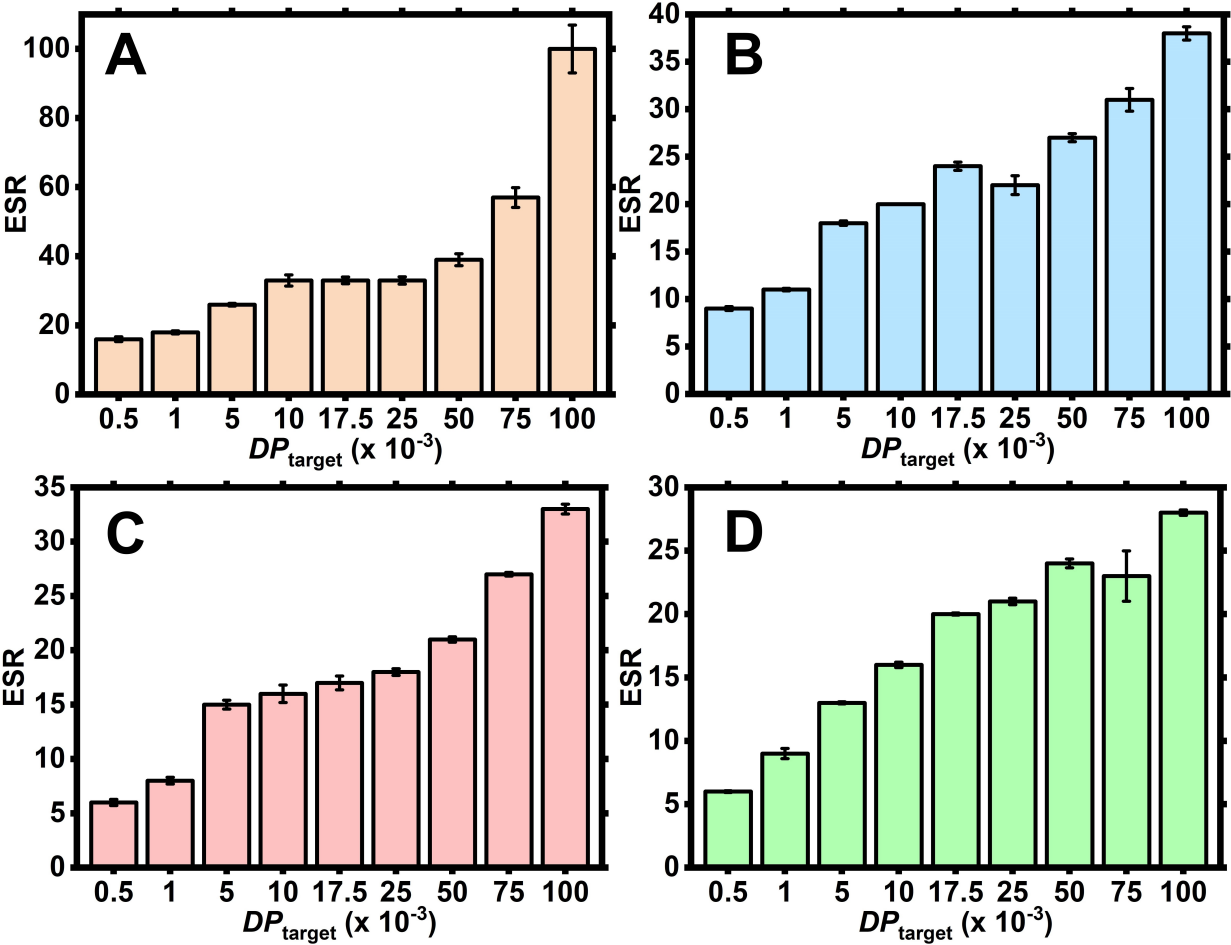
ESR (H_2_O) data for UHMW PDMAm hydrogels with [MBAm] : [CTA]=2 (**A**), 5 (**B**), 8 (**C**) and 10 (**D**). All measurements were performed in triplicate.

**Hydrogel swelling and degradation**. Next, the equilibrium swelling ratio (ESR) was determined by allowing the hydrogels to swell in water for 48 h and calculated as ESR=*m*_swollen_/*m*_dry_ (Figure 4). As expected, the ESR of all hydrogel series, regardless of the initial crosslinker content, increased as the *DP*_target_ of PDMAm increased.

However, a clear plateau for the ESR of each [MBAm] : [CTA] ratio can be observed at *DP*_target_ between c.a. 10,000–50/75,000. This suggests that even though the crosslinking density is greatly decreased, physical entanglements are preventing the hydrogel from swelling more as they begin to dominate over crosslinks. This trend is then broken and the hydrogels with *DP*_target_ of 100,000, which have extremely low crosslinking densities, (i.e. molar ratio between 2×10^−5^ and 10^−4^ vs monomer) start to disentangle and swell more but do not dissolve. A similar swelling behaviour of the PDMAm hydrogels was also observed in methanol (Figure S6).

In order to gain more insight about the internal structure of the entangled hydrogels, a series of UHMW PDMAm hydrogels crosslinked with a cleavable crosslinker (*N*,*N*’‐bis(acryloyl)cystamine, BAC), were synthesized with [BAC] : [CTA]=10. The resulting hydrogels contain disulfide bonds which, when exposed to thiol‐containing degradation agents, should be reduced by thiol‐disulfide exchange.[^39^, ^40^, ^41^, ^63^] To test this, samples of the hydrogels were immersed in a solution of dithiothreitol (DTT) in DMF (25 mg/mL) at 65 °C to initiate degradation.

**Figure 5:**
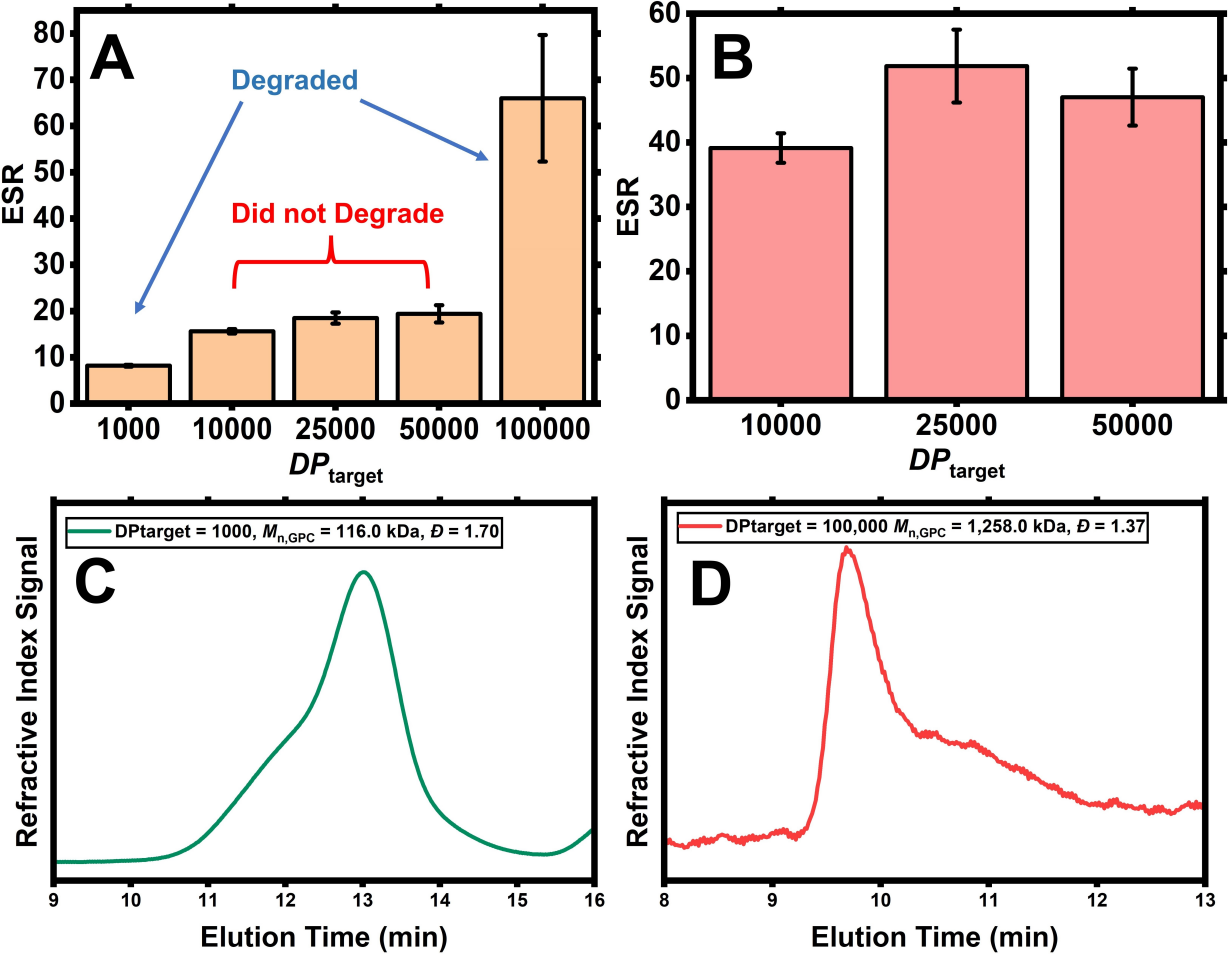
ESR (H_2_O) pre‐degradation (**A**) and post‐degradation (**B**) of UHMW PDMAm‐BAC hydrogels. All measurements were performed in triplicate. GPC traces for the degraded gel fragments of the PDMAm‐BAC hydrogels with *DP*_target_ of 1000 (**C**) and 100,000 (**D**).

The ESR values for the PDMAm‐BAC hydrogels follow a similar pattern to their PDMAm‐MBAm analogues whereby the values plateau at high *DP*_target_ before significantly increasing at vanishingly low crosslinking densities (Figure 5A). Interestingly, the hydrogels on this ESR plateau (i.e., *DP*_target_=10,000–50,000) did not exhibit macroscopic degradation when exposed to excess DTT/DMF for 40 days. The remaining, swollen gel fragments were washed with water, dried and re‐swollen in water yielding much higher ESR values (Figure 5B). On the other hand, the hydrogels with *DP*_target_ of 1000 and 100,000 displayed full macroscopic degradation within the first 24 h and samples of their degraded fragments were analysed by gel permeation chromatography (GPC, Figure 5C and 5D, respectively). The sample with *DP*_target_=1000 had *M*_n, GPC_=116,000 g mol^−1^ which is close to the theoretical value at full conversion (*M*_n, theo_=99,130 g mol^−1^). There is some evidence of branching visible as high MW tailing with *Ð*=1.70, likely caused by some non‐cleaved crosslinks. Degradation of the *DP*_target_=100,000 hydrogel produced fragments with *M*_n_>1,200,000 g mol^−1^ although this value is not fully accurate as it was partially beyond the calibration range of the instrument. Nevertheless, relatively low *Ð*=1.37 was observed, indicating that the polymerization was still controlled even at such a high *DP*_target_.

The results from the degradation experiments were surprising as the gels made by RDRP techniques with cleavable crosslinkers are commonly observed to fully degrade due to their homogeneous internal network structures imposed by controlled polymerization, leaving crosslinks exposed when swollen.[^39^, ^40^, ^41^, ^64^] Therefore, the interplay between high density of entanglements and low, but sufficient density of crosslinks to prevent chains from disentangling upon swelling likely impedes degradation by not allowing the hydrogel to swell more. At the extremely reduced crosslinking density (*DP*_target_=100,000), the network strands start to disentangle, the hydrogel continues to swell and eventually degrades.

A control PDMAm‐BAC hydrogel prepared using conditions that would be similar to a *DP*_target_ of 25,000 was synthesized by conventional FRP with VA‐044 as an initiator in the absence of a RAFT agent. The hydrogel yielded an ESR of 5.58 ±0.57, did not degrade after 40 days exposure to the DTT/DMF solution and had a post‐degradation ESR of 7.00±0.73. However, degradation was not expected to occur in this case due to the spatial inhomogeneities, such as non‐swellable nanoclusters, that arise during FRP synthesis of polymer gels.[^33^, ^65^] This is in contrast to a recent work from Suo *et al*., who reported full degradation of highly entangled polyacrylamide hydrogels synthesized by FRP with degradable crosslinks.^[28]^ Likely, the differences in polymerization kinetics between acrylamide and DMAm monomers lead to a larger extent of intramolecular cyclization in the former case, resulting in hydrogels with reduced effective crosslinking density, and therefore more susceptibility to degradation.[^33^, ^66^] Nevertheless, in RDRP (especially at low crosslinker loadings) formation of spatial inhomogeneities is suppressed and the hydrogel maintains its well‐defined structure. Thus, restricted swelling and degradation can be ascribed exclusively to the presence of dense entanglements and minimal, but sufficient number of crosslinks to prevent disentangling.

**Initial monomer concentration**. Finally, the effect of dilution on the formation of highly entangled hydrogels and their resultant physical properties was examined. The initial monomer concentration, [M]_0_, and the resulting polymer volume fraction *φ* will affect the onset of entanglement as evident from eq. 2. Previous reports on polyacrylamide hydrogels used [M]_0_ varying from 1.0–5.0 M^[23]^ up to unusually high values (i.e. 28 M, note that bulk concentration of DMAm=9.70 M).^[22]^ Thus, to test how the structural properties of the PDMAm hydrogels differ depending on the [DMAm]_0_, a series of PDMAm hydrogels with varying [DMAm]_0_ were prepared.

**Figure 6:**
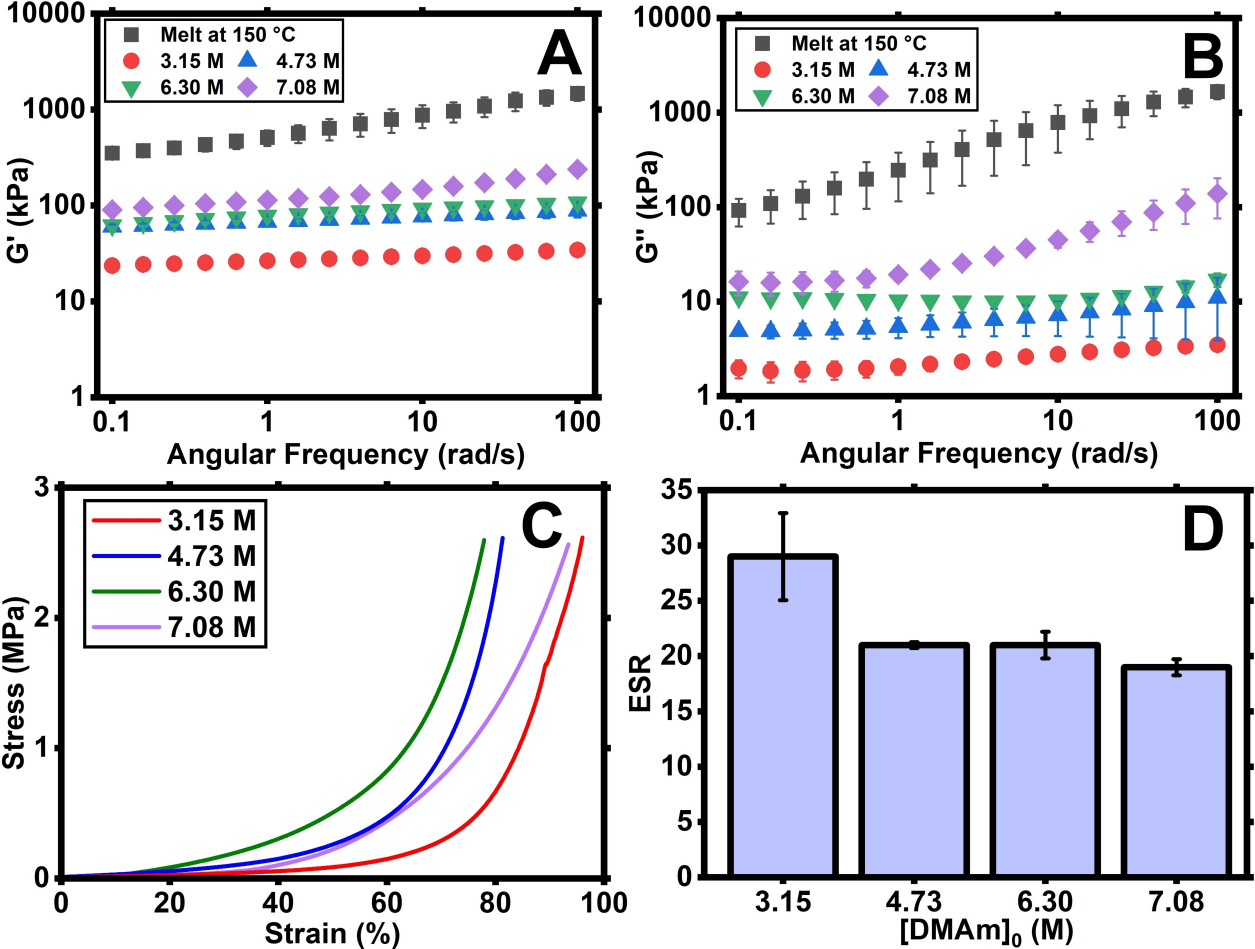
Results from the analysis of UHMW PDMAm hydrogels formed with varying [DMAm]_0_ while maintaining [MBAm] : [CTA]=10 and a *DP*_target_ of 25,000, compared with uncrosslinked PDMAm melt at 150 °C (black squares). Shown are storage moduli data **(A)**, loss moduli data (**B)**, compression stress‐strain curves **(C)**, and ESR (H_2_O) data **(D)** for all concentrations. All swelling and rheological measurements were performed in triplicate.

PDMAm hydrogels with [MBAm] : [CTA]=10 and a *DP*_target_ of 25,000 (which was firmly on the modulus plateau shown in Figure 1B **)** were examined. Oscillatory rheology measurements were conducted as outlined previously for each dilution of PDMAm hydrogel. Figure 6A shows shear storage moduli for the four different dilutions and uncrosslinked melt at 150 °C. As expected, the hydrogels with higher [DMAm]_0_ have slightly higher values for *G*’ due to the higher concentration of elastically active strands and more entanglements. The hydrogels with [DMAm]_0_=3.15–6.30 M showed no frequency dependence and phase angles <10° indicating typical elastic behaviour, however the sample with the highest [DMAm]_0_=7.08 M (i.e., *φ*=0.73 assuming full conversion) exhibited more frequency dependence and increased phase angles at higher frequencies, similar to the uncrosslinked melt at 150 °C (Figures 6A, B and S7).

Compressive stress/strain testing was carried out on the samples and their results are shown in Figure 6C and Movies S4 and S6–S8. These results show that for all [DMAm]_0_, the hydrogels did not break under compression and exhibited high compressive strength with maximum stress value >2.50 MPa in all cases, indicative of high toughness resulting from dense entanglements and low crosslinking density. This suggests that once the *DP*_target_ is sufficiently high to ensure *M*_n_>*M*_c,sol_, which is the case for all tested formulations, a highly entangled hydrogel can be formed. However, the polymer volume fraction will affect at what moduli values the plateau is observed, tending towards the limiting value for melt at higher *φ*. ESR values (Figure 6D) remain relatively constant regardless of [DMAm]_0_, with the lowest [DMAm]_0_ (3.15 M) having the highest ESR (30) and the more concentrated samples displaying similar values (19–21), which is expected as they have the same overall crosslinking density (i.e., [MBAm] : [CTA]=10). These results suggest that high concentration of monomer/polymer is not itself critical for the formation of highly entangled hydrogels. The main prerequisite is to ensure that *M*_n_>*M*_c,sol_ and a sufficiently high *M*_x_ are achieved to make the crosslinks’ contribution to the strength of the hydrogels negligible.

## Conclusion

Highly entangled PDMAm hydrogels with UHMW primary chains were synthesized using photoiniferter‐mediated polymerization. A simple approach to control both the entanglements and crosslinking density by varying *DP*_target_ at a fixed crosslinker/photoiniferter ratio was introduced. It was shown that once *M*_n_ of the primary chains exceeds critical molecular weight of entanglements in solution, and the crosslinking density was sufficiently diminished, a plateau appears in storage modulus values for hydrogels with a *DP*_target_ over 5,000 and [MBAm] : [CTA]=10. This suggests that at higher *DP*_target_, physical entanglements are exclusively responsible for the strength of the hydrogel while the crosslinking density is vanishingly low but enough to maintain its structural integrity. The hydrogels whose modulus is on the plateau were shown to have greatly improved physical properties, namely resistance to compression, swelling and chemical degradation if prepared with cleavable crosslinks.

Utilizing controlled polymerization methods to synthesize entangled hydrogels has practical advantages such as simplicity and low cost (i.e. minute amounts of the photoiniferter), while providing a more uniform internal structure devoid of spatial and topological defects associated with free radical polymerization. This allows for a better understanding of the influence of entanglements on hydrogels’ properties, while the ability to control *M*_n_ should enable more routine preparation of gels/networks in the entangled regime. We believe that this study presents both the facile synthetic method and more comprehensive understanding of highly entangled hydrogels and will streamline further work on their emerging applications, such as impact resistance or shock absorption, which require materials with improved energy dissipation.

## Supporting Information

Experimental details, gel fractions, additional rheology and swelling data, determination of *M*_e_ for uncrosslinked PDMAm, compression test images and movies.

## Conflict of Interests

The authors declare no conflict of interest.

1

## Supporting information

As a service to our authors and readers, this journal provides supporting information supplied by the authors. Such materials are peer reviewed and may be re‐organized for online delivery, but are not copy‐edited or typeset. Technical support issues arising from supporting information (other than missing files) should be addressed to the authors.

Supporting Information

Supporting Information

Supporting Information

Supporting Information

Supporting Information

Supporting Information

Supporting Information

Supporting Information

Supporting Information

## Data Availability

The data that support the findings of this study are available from the corresponding author upon reasonable request.
