# Supplementary material for: Highly Entangled Hydrogels by Photoiniferter‐Mediated Polymerization
Source: Angew Chem Int Ed Engl. 2025 Feb 21;64(17):e202421970. doi: 10.1002/anie.202421970 (PMC12015391; doi:10.1002/anie.202421970)
Supplement: Supplementary file 1 — Supporting Information [file ANIE-64-e202421970-s006.pdf]

Angewandte Chemie
Eine Zeitschrift der Gesellschaft Deutscher Chemiker

Supporting Information

# Highly Entangled Hydrogels by Photoiniferter-Mediated Polymerization

*G. Irvine, K. Myronidis, F. Pinto, M. Kopeć**

Supporting Information

# Highly Entangled Hydrogels by Photoiniferter-Mediated Polymerization

*Gavin Irvine,[a] Konstantinos Myronidis,[b] Fulvio Pinto,[b] Maciej Kopeć* [a]*

[a]Department of Chemistry, University of Bath, Claverton Down, Bath BA2 7AY, UK

[b]Department of Mechanical Engineering, University of Bath, Claverton Down, Bath BA2 7AY, UK

*e-mail: mk2297@bath.ac.uk

## Experimental

### Materials

*N*,*N*'-Bis(acryloyl)cystamine (BAC, 98%) was purchased from Alfa Aesar. Dithiothreitol (DTT) was purchased from Thermo Scientific. *N*,*N*'-Methylenebisacrylamide (MBAm, 99%), *N*,*N*-dimethylacrylamide (DMAm, 99%), lithium bromide, *N*,*N*-dimethylformamide (DMF, ≥99.9%), and 2-cyano-2-propyl dodecyl trithiocarbonate were purchased from Sigma-Aldrich. 2,2'-Azobis[2-(2-imidazolin-2-yl)propane] dihydrochloride (VA-044) was purchased from FUJIFILM Wako Pure Chemical Corporation. To remove inhibitor, DMAm was passed through a column of basic alumina before use.

### General synthesis of UHMW PDMAm polymer gels by photoiniferter polymerization

In a typical experiment targeting a degree of polymerization ($DP_{target}$) of 10,000 for PDMAm, DMAm (2 mL, 18.90 mmol), 2-cyano-2-propyl dodecyl trithiocarbonate (0.26 mg, 0.002 mmol), water (2 mL) and the acrylamide crosslinker, MBAm (1.17 mg, 0.02 mmol) or BAC (5.21 mg, 0.02 mmol), were mixed in a neckless glass vial. The vial was equipped with a rubber septum and degassed with nitrogen for 20 min before being placed under a nail lamp, irradiated with UV light at $\lambda_{max}$ = 365 nm and left for 6 h.

### Synthesis of a UHMW PDMAm-BAC polymer gel by FRP

DMAm (2 mL, 18.90 mmol), VA-044 (0.24 mg, 0.0008 mmol), water (2 mL) and the N,N'-bis(acryloyl)cystamine crosslinker, BAC (2.00 mg, 0.008 mmol), were mixed in a neckless glass vial. The vial was equipped with a rubber septum and degassed with nitrogen for 20 min before being placed under a nail lamp and irradiated with UV light at $\lambda_{max}$ = 365 nm for 6 h.

### Equilibrium swelling ratio

Swelling was performed by immersing an as-synthesized piece of gel (40-100 mg) in excess deionized water for 48 h. Any excess surface solvent was removed before recording the mass of the swollen gel. The gel pieces were then dried in a vacuum oven for up to 48 h and the mass of the dried gel was recorded. The equilibrium swelling ratio (ESR) was calculated as ESR = $m_{swollen}/m_{dry}$ with all measurements being performed in triplicate.

### Determination of gel fraction

40-100 mg of a fully formed hydrogel sample (after 6 h reaction) was placed in a vial with 3 mL of methanol and left for at least 24 h. The methanol was then transferred to a separate, pre-weighed vial and allowed to evaporate overnight in a fume cupboard. The dried vial was

reweighed to determine the mass of the remaining solids ($m_{sol}$); this washing cycle was repeated at least three times until a constant mass was achieved. The washed gel was dried under vacuum overnight and weighed to determine $m_{gel}$. The final gel fraction was calculated as %GF = $m_{gel}/(m_{gel} + m_{sol}) \times 100\%$. For gel fraction analysis, all measurements were performed in triplicate.

**Degradation of UHMW PDMAm-BAC hydrogels**

40-100 mg of a fully formed hydrogel sample (after 6 h reaction) was placed in a vial with 3 mL of DTT in DMF (25 mg $mL^{-1}$) and left at 65 °C for up to 40 days. If macroscopic degradation occurred (i.e. hydrogel sample dissolved), the vial was opened, and the soluble degradation products were analysed by gel permeation chromatography (GPC). If macroscopic degradation did not occur, the remaining swollen gel fragments were washed with deionized water to remove the DTT and DMF, dried under vacuum overnight and re-swollen in water. All measurements were performed in triplicate.

**Compression testing on as-synthesized hydrogels**

Quasi-static compressive tests were performed on the as-synthesized cylindrical hydrogel samples using an Instron Universal Test Frame 3369. Each hydrogel sample was characterized by a diameter between 21 – 23 mm and by a thickness between 4 – 10 mm. The moving crosshead of the testing machine was equipped with a 1 kN load cell and the samples were tested at a compression rate of 1 mm/min (adapted from ASTM D695). Compressive stress was evaluated at each point by dividing the compressive load carried by the specimen during the test by its original cross-sectional area, while compressive strain was calculated by dividing the compressive displacement by the original thickness of the sample. Energy absorption diagrams were evaluated by calculating the energy absorbed by each sample during the compressive tests (according to $E = \int_0^{\varepsilon} \sigma d\varepsilon$) and plotting it as a function of the applied stress.

**Instrumentation**

GPC analysis of PDMAm samples was performed on Agilent 1260 Infinity fitted with an autosampler and refractometer. The system used Polargel-M 300 × 7.5 mm columns. Analysis was carried out at 40˚C with lithium bromide (10 mmol) in DMF as the eluent. The sample injection was 50 µL with a run time of 30 min and a 1 mL/min flow rate. Data acquisition was performed using linear poly(methyl methacrylate) as the calibration standard. Oscillatory rheology measurements were carried out using a TA Instruments Discovery HR-3 rheometer fitted with a 20 mm crosshatched parallel plate geometry and a crosshatched base plate. As-

synthesized disk-shaped gel samples were assessed under a constant axial force of 4 N. Frequency sweeps were carried out at 25 ˚C over a range of 0.1-100 rad$^{-1}$ at a constant strain of 1%. All rheological measurements were performed in triplicate. For oscillatory rheology measurements to determine $M_e$ of PDMA, the same rheometer was fitted with a 20 mm crosshatched parallel plate geometry and a crosshatched base plate. Dried disk-shaped melt sample with a thickness of c.a. 7 mm were assessed under a constant axial force of 0.5 N. Frequency sweeps were carried out between 150 – 190 ˚C over a range of 0.01-100 rad$^{-1}$ at a constant strain of 1%. Shift factor analysis was subsequently conducted as outlined by Ricarte and Shanbhang[1] to obtain the frequency sweep master plot (**Figure S3**) for the determination of $M_e$.

**Table S1**: Gel fractions (%, MeOH) of the PDMAm hydrogels with [MBAm]:[CTA] = 10 and $DP_{target}$ = 500 – 100,000.

| $DP_{target}$ | **Gel Fraction (%)** |
|---|---|
| 500 | 99.39 ±0.51 |
| 1000 | 98.54 ±0.94 |
| 5000 | 98.75 ±0.03 |
| 10,000 | 98.03 ±0.29 |
| 17,500 | 98.29 ±0.30 |
| 25,000 | 97.52 ±0.97 |
| 50,000 | 96.78 ±0.75 |
| 75,000 | 97.28 ±0.58 |
| 100,000 | 95.81 ±1.21 |

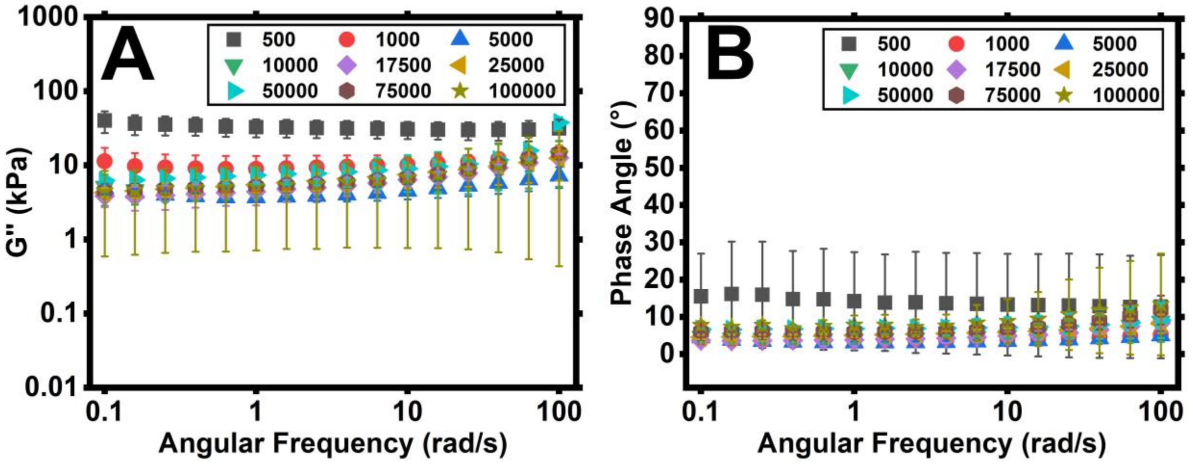


**Figure S1**: Loss moduli data (*G''*, kPa, **A**) and phase angles (°, **B**) for UHMW PDMAm hydrogels with [MBAm]:[CTA] = 10. All measurements were performed in triplicate.

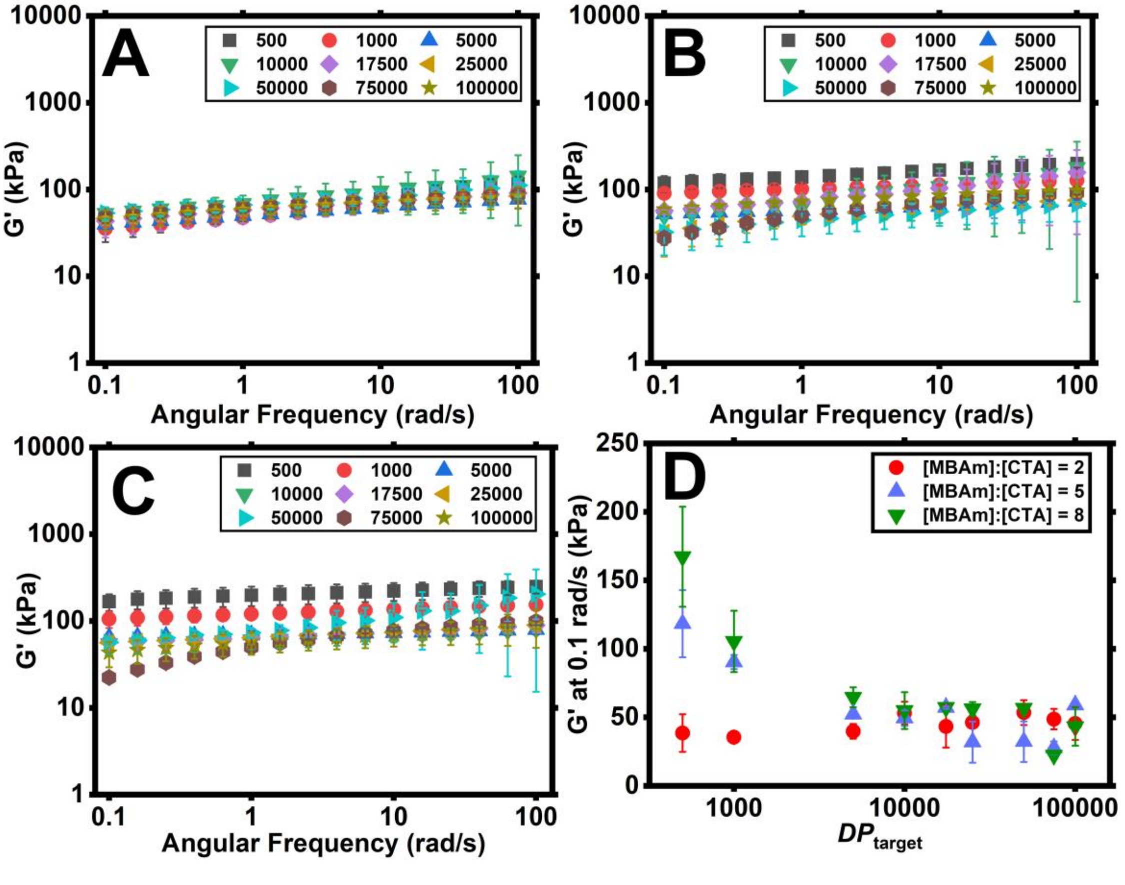


**Figure S2**: Storage moduli (*G'*, kPa) data for UHMW PDMAm gels with [MBAm]:[CTA] = 2 (**A**), 5 (**B**), and 8 (**C**). The *G'* values at 0.1 rad/s plotted vs $DP_{target}$ are for all [MBAm]:[CTA] ratios (**D**). All measurements were performed in triplicate.

**Determination of the entanglement molecular weight ($M_e$) of PDMAm**

In order to obtain a value for the entanglement molecular weight ($M_e$) of PDMAm, a sample of PDMAm with a $DP_{target}$ of 25,000 was synthesized without a crosslinker. This $DP_{target}$ was chosen as its hydrogel analogue with [MBAm]:[CTA] = 10 lay firmly on the modulus plateau observed in **Figure 1B**, suggesting the effect from entanglements on modulus has overtaken the effects of crosslinking at this point. The linear PDMAm sample was synthesized following the same procedure as its hydrogel analogue (50% v/v $H_2O$, λ = 365 nm, 6 h) but without the addition of MBAm. The sample was formed so that it would have a disc shape and after synthesis, it was dried in a vacuum oven overnight. Subsequently, oscillatory frequency sweeps were conducted at 150-190 °C after equilibrating the sample for 10 min (**Figure S3**). The $M_e$ value was then determined from equation S1[2] by taking the *G'* value with the lowest tan delta ($G_n$ = 266,787 Pa at 0.00349 rad/s), $\rho$ = 1090 kg/m$^3$ at $T_g$,[3] R = 8.314 J mol$^{-1}$ K$^{-1}$ and T = 423.15 K. This yielded a $M_e$ value of 15,400 g mol$^{-1}$ which is within the typically observed range for acrylic polymers.

$$G_n = \frac{\rho R T}{M_e} \tag{S1}$$

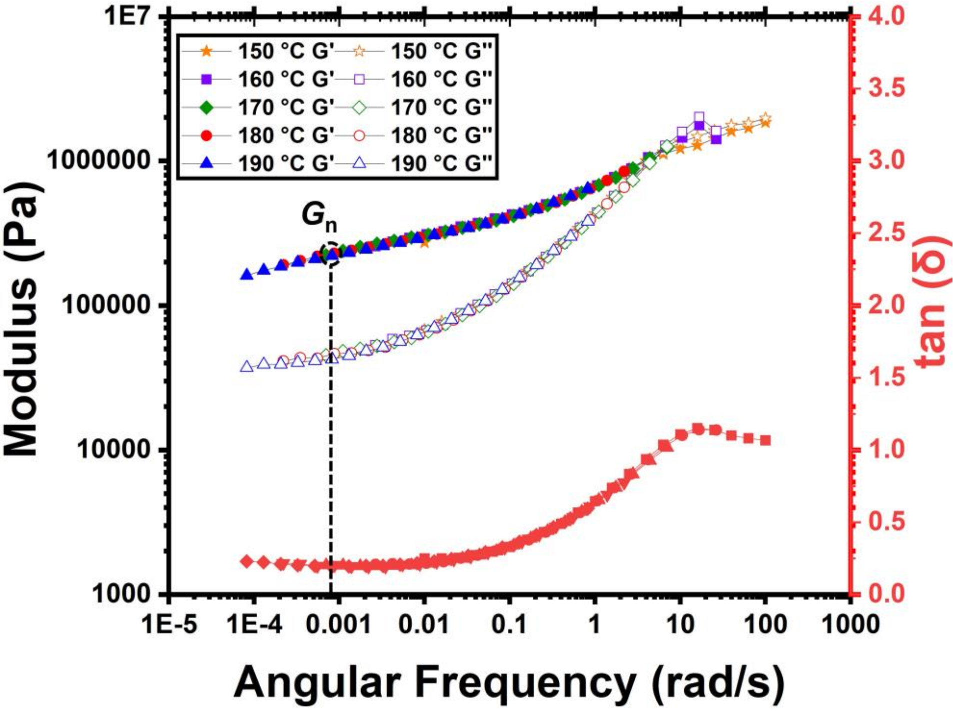


**Figure S3**: Frequency sweep master plot showing the storage (*G'*) and loss (*G''*) moduli values (Pa) for a dried disc of UHMW PDMAm with a $DP_{target}$ of 25,000 and no crosslinker present for determination of $G_n$ to be used in the calculation of $M_e$.

**Compression tests of PDMAm gels of different *DP*target.**

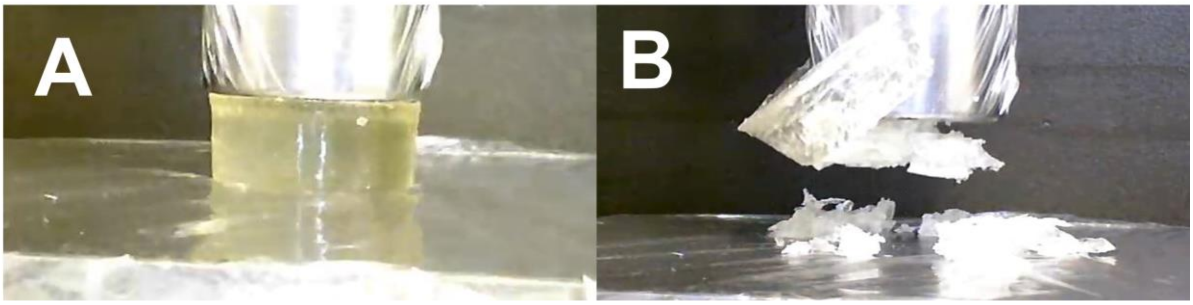


**Figure S4**: Pictures of a PDMAm gel with a $DP_{\text{target}}$ of 1000 both prior to (**A**) and after (**B**) compression tests.

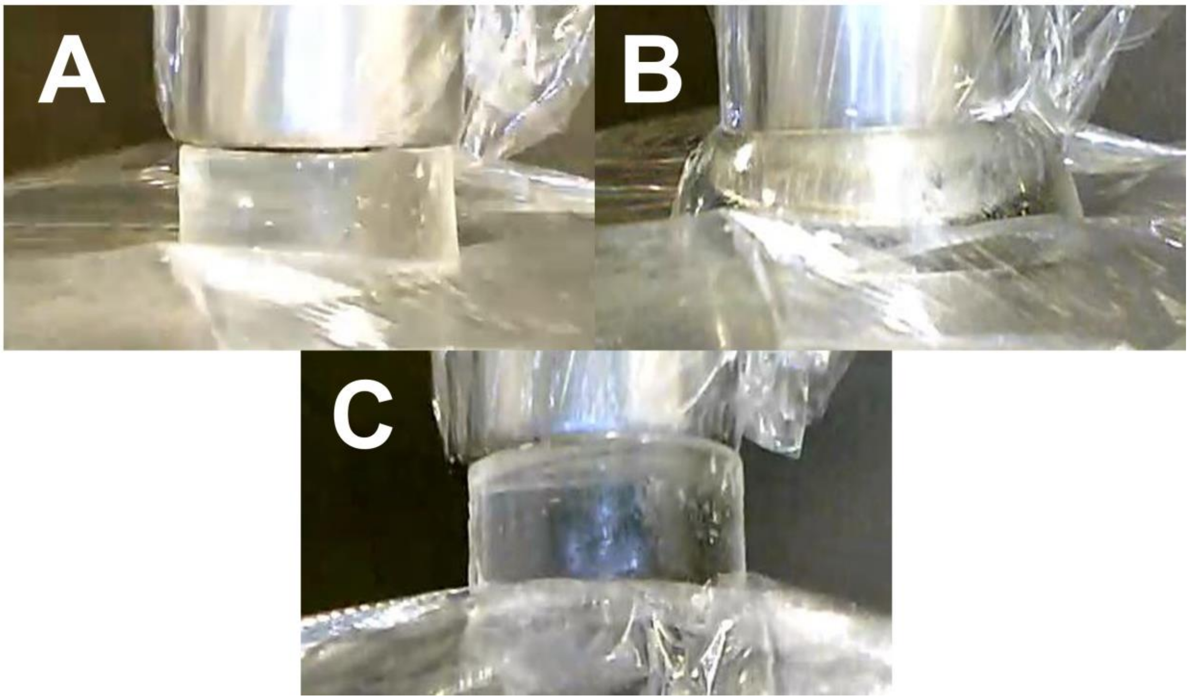


**Figure S5**: Pictures of a PDMAm gel with a $DP_{\text{target}}$ of 25,000 prior to compression testing (**A**), at full displacement (**B**) and after (**C)** compression tests.

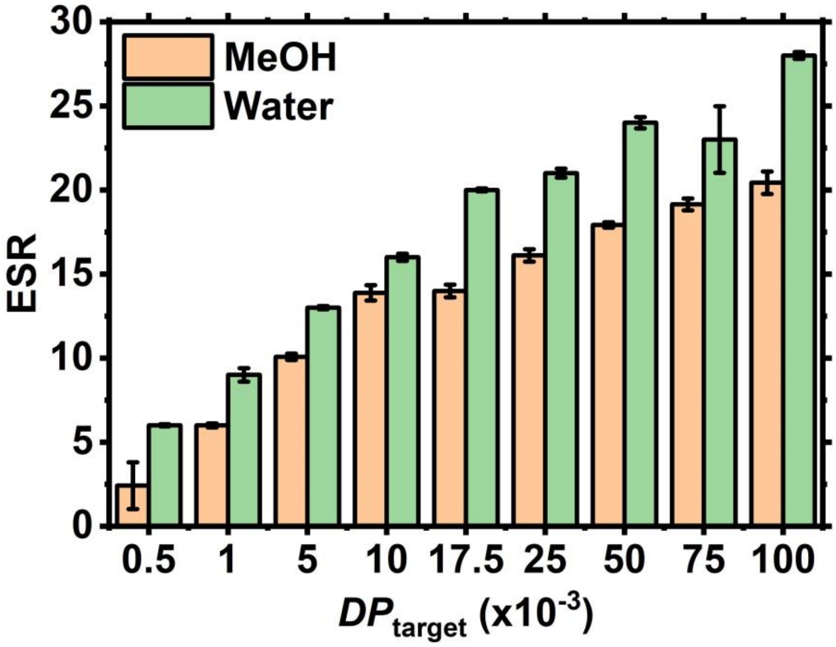


**Figure S6**: ESR comparison of the PDMAm hydrogels with [MBAm]:[CTA] = 10 and various $DP_{target}$ conducted in water or methanol. All measurements were performed in triplicate.

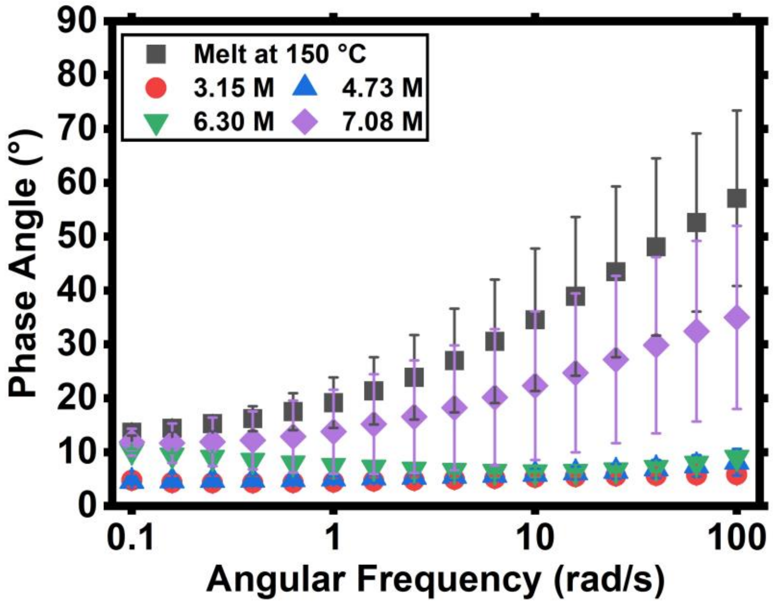


**Figure S7**: Phase angles (°) for the PDMAm hydrogels with a $DP_{target}$ of 25,000 and varying $[DMAm]_0$ as well as for the uncrosslinked melt at 150 °C. All measurements were performed in triplicate.

## References


(1) G. Ricarte, R.; Shanbhag, S. A Tutorial Review of Linear Rheology for Polymer Chemists: Basics and Best Practices for Covalent Adaptable Networks. *Polymer Chemistry* **2024**, *15* (9), 815–846. https://doi.org/10.1039/D3PY01367G.

(2) Lessard, J. J.; Stewart, K. A.; Sumerlin, B. S. Controlling Dynamics of Associative Networks through Primary Chain Length. *Macromolecules* **2022**, *55* (22), 10052–10061. https://doi.org/10.1021/acs.macromol.2c01909.

(3) Jones, E. R.; Mykhaylyk, O. O.; Semsarilar, M.; Boerakker, M.; Wyman, P.; Armes, S. P. How Do Spherical Diblock Copolymer Nanoparticles Grow during RAFT Alcoholic Dispersion Polymerization? *Macromolecules* **2016**, *49* (1), 172–181. https://doi.org/10.1021/acs.macromol.5b02385.
